# Demonstration of autonomous fixed‐wing drone delivery of prescribed medications in remote islands in Japan

**DOI:** 10.1002/jgf2.768

**Published:** 2025-01-09

**Authors:** Jun Miyata, Hironobu Tsuchiya, Fumiaki Nonaka, Yuji Aso, Masanori Sugahara, Takahiro Maeda

**Affiliations:** ^1^ Department of Island and Community Medicine Nagasaki University Graduate School of Biomedical Sciences Nagasaki Japan; ^2^ Sora‐iina Corporation Nagasaki Japan; ^3^ Hisaka Clinic Nagasaki Japan; ^4^ Fukue Pharmacy Nagasaki Japan; ^5^ Department of General Medicine Nagasaki University Graduate School of Biomedical Sciences Nagasaki Japan; ^6^ Leading Medical Research Core Unit Nagasaki University Graduate School of Biomedical Sciences Nagasaki Japan

**Keywords:** distance counseling, drone, drug, pharmacy, rural health services, telemedicine

## Abstract

**Background:**

In rural areas, physicians and dentists often dispense medications without pharmacists. Technology that can safely and promptly deliver medications is required so that pharmacists, even those at a distance, can dispense them in a timely manner.

**Methods:**

We demonstrated a combination of online medication counseling and drone delivery of medications dispensed by pharmacists in two rural clinics located far from pharmacies on remote islands in Japan. After being prescribed by physicians, dispensed by pharmacists, and online medication counseling, the medications were transported by autonomous fixed‐wing drones, dropped, and picked up by the staff. Following Japanese regulations, our drones could only fly over the sea and drop medications on the shore. After the demonstration, we administered a questionnaire.

**Results:**

A total of 62 patients participated in the demonstration, which was successful except for two patients whose drone deliveries were canceled because of strong winds. Most patients received their medications after more than an hour. Individual home delivery costs were involved because the delivery spot was restricted to the shore. More than 80% of the patients were satisfied with online medication counseling, but a few expressed the desire to receive their medication at the clinic.

**Conclusions:**

Prescribed medication delivery using drones was technically feasible. This project will contribute to separating the prescribing and dispensing functions, promote pharmacist participation in rural areas, and expand patient treatment options. Methods for checking and monitoring the delivery spot, ensuring successful delivery, and covering the cost of drone delivery need further exploration.

## Graphical abstract

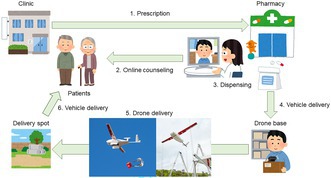

## 1 INTRODUCTION

Many countries have implemented a government policy to separate prescribing and dispensing practices.^1^ Under this policy, residents who want to obtain the ethical medications submit a received prescription from physicians or dentists to pharmacists, who review prescriptions, provide medical counseling, and may inquire with prescribers (physicians and dentists) if needed. This policy would reduce the misuse and overuse of medications, leading to the patients' safety and therapy effectiveness,^1^, ^2^, ^3^, ^4^, ^5^ as well as conflict of interest management.^1^, ^2^, ^3^ In Japan, the nationwide average rate of separation of prescribing and dispensing functions has increased owing to the government's dispensing separation policy: 12% in 1990, 40% in 2000, 63% in 2010, and 75% in 2019.^6^ Residents cannot obtain medication without a prescription, except for some over‐the‐counter medications, used for common symptoms and disorders that are easily recognizable by an ordinary consumer (e.g., cold medicines, anti‐inflammatory analgesic agents, and gastrointestinal drugs).^7^

Nevertheless, physicians and dentists often dispense medications in many rural areas without pharmacists. In Japan, more than 90% of the medical facilities in rural areas where medications are possibly dispensed do not have a pharmacist.^8^ In such local medical facilities, patients are forced to choose from limited treatment options because these facilities are limited in medicines, especially in sparsely populated areas, in addition to the disadvantages mentioned above. Alternatively, patients can receive medications dispensed by pharmacists at a distance by mail after online medication counseling. In Japan, online medication counseling became permitted in April 2020 as a countermeasure against the coronavirus 2019 (COVID‐19) pandemic and was institutionalized in the Act on Securing Quality, Efficacy and Safety of Products Including Pharmaceuticals and Medical Devices^9^ in September 2020. However, although road infrastructure is well developed in Japan, mail delivery is time‐consuming (not available for same day delivery) and incurs a cost (e.g., 600 Japanese yen for “Letter Pack,” which is a face‐to‐face delivery service by Japan Post^10^). Additionally, it is desirable to avoid burdening the ground vehicle logistics industry to address a shortage of truck drivers, which is estimated to reach 34% by 2030.^11^ Technology is required to deliver medications safely and promptly, with less effort, so that pharmacists, even those at a distance, can dispense them in a timely manner.

Drone technology is broadly applied to the delivery of medications, blood products, and automated external defibrillators worldwide,^12^, ^13^ and has the potential to solve this problem. In Japan, Sora‐iina Corporation uses fixed‐wing drones for delivery logistics in the Goto Islands,^14^ while Amami Island Drone Co., Ltd. uses a single‐rotor drone in the Amami Islands.^15^ Other companies, such as Aeronext Inc., use multi‐rotor drones, mainly in rural areas.^16^ Drone technology may allow the quick transport of medications dispensed by pharmacists to rural areas and remote islands across the sea.

Therefore, we launched a project to develop a medical service combining online medication counseling and autonomous fixed‐wing drone delivery of prescribed medications. We conducted a demonstration on remote islands in Japan to examine the feasibility and limitations of its practical applications.

## 2 METHODS

### 2.1 Demonstration setting and participants

**FIGURE 1:**
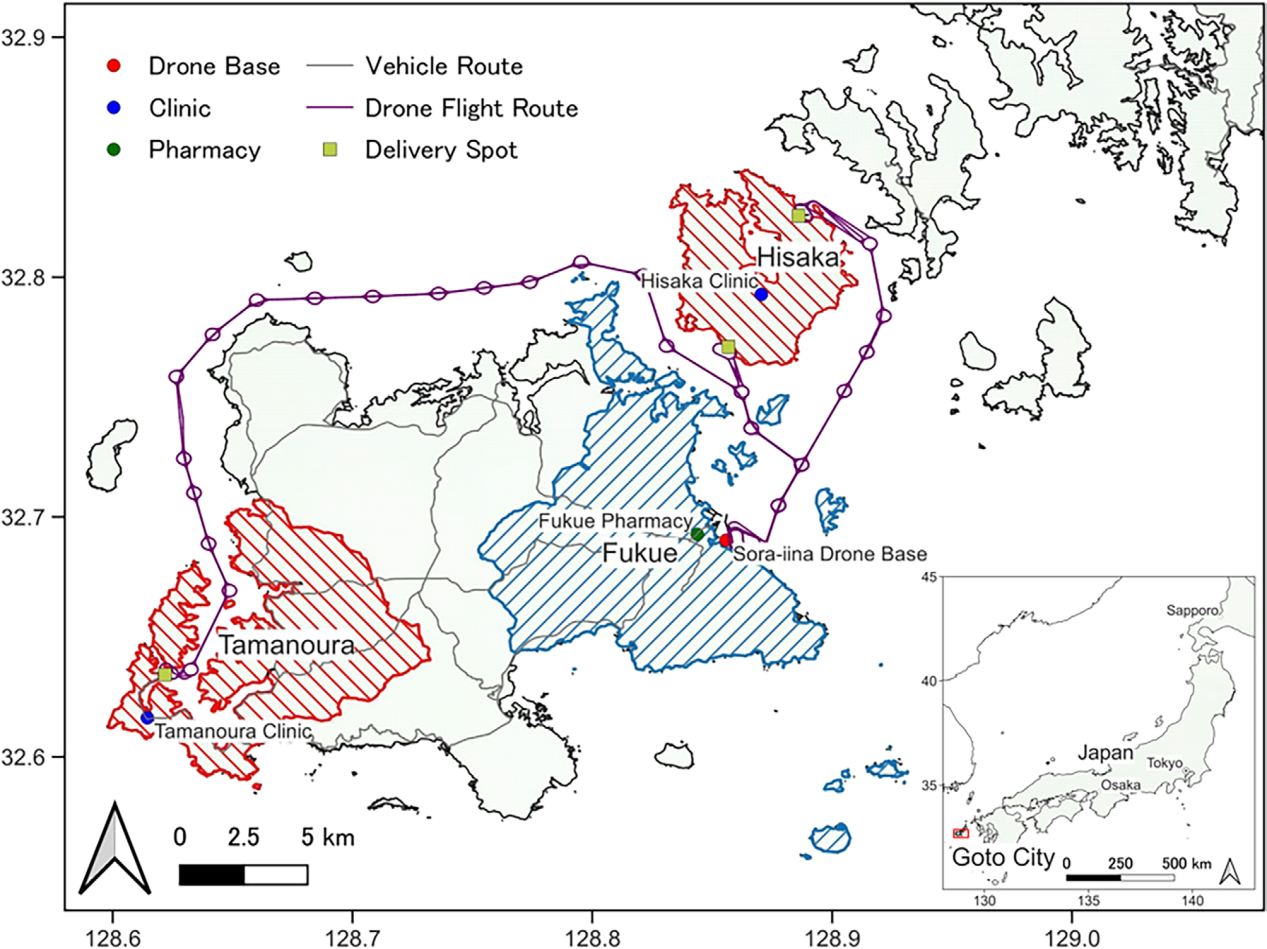
Map of the target areas of this demonstration, the Hisaka Island and the Tamanoura District in Goto City, where there are public clinics (Hisaka Clinic and Tamanoura Clinic, respectively) but no pharmacists or pharmacies with a small population. In the Fukue District, the drone base is operated by Sora‐iina Corporation, and many pharmacies are located.

We demonstrated a medical service that combines online medication counseling and drone delivery of medications dispensed by pharmacists in Goto City, located on the remote islands of Nagasaki Prefecture, Japan (Figure 1). The target areas of this demonstration were Hisaka Island and the Tamanoura District on Fukue Island, where there are public clinics (Hisaka Clinic and Tamanoura Clinic, respectively) but no pharmacists or pharmacies with a small population. As of October 2023, the population was 266 (59.0% were ≥65 years old) on Hisaka Island and 1167 (58.0% were ≥65 years old) in Tamanoura District. Telemedicine is not widely available in these areas. In Fukue District, where the population of Fukue Island is concentrated, the drone base is operated by Sora‐iina Corporation, and many pharmacies are located.

We recruited patients and their families who visited Hisaka Clinic between October 3 and November 2, 2023, or Tamanoura Clinic on November 6, November 20, December 4, and December 18, 2023, and were prescribed medications. Of these, we excluded patients prescribed narcotics, some psychotropic medications, and toxic medications, which were not allowed to be delivered by drone under the guidelines as of March 2023.^17^ We invited all patients who did not meet the exclusion criteria to participate in this study for Hisaka Clinic, and at Tamanoura Clinic, we asked ~10 patients per day to participate, without specific selection criteria, owing to the high volume of daily patients. The participants were paid 2000 Japanese yen as a cooperation fee. For this demonstration, we asked Fukue Pharmacy (one of the pharmacies in Fukue District) to cooperate by dispensing medications and providing online medication counseling to patients.

The protocol for this demonstration was approved by the Ethics Committee of Nagasaki University Graduate School of Biomedical Sciences (project registration number: 23072804). This demonstration was conducted in accordance with the ethical standards of the 1964 Declaration of Helsinki and its subsequent amendments. This demonstration was registered with the UMIN Clinical Trials Registry (UMIN000052074). All participants provided written informed consent, and most had completed it ~1 month before the demonstration.

### 2.2 Flow of prescribing, online medication counseling, dispensing, and medication delivery

**FIGURE 2:**
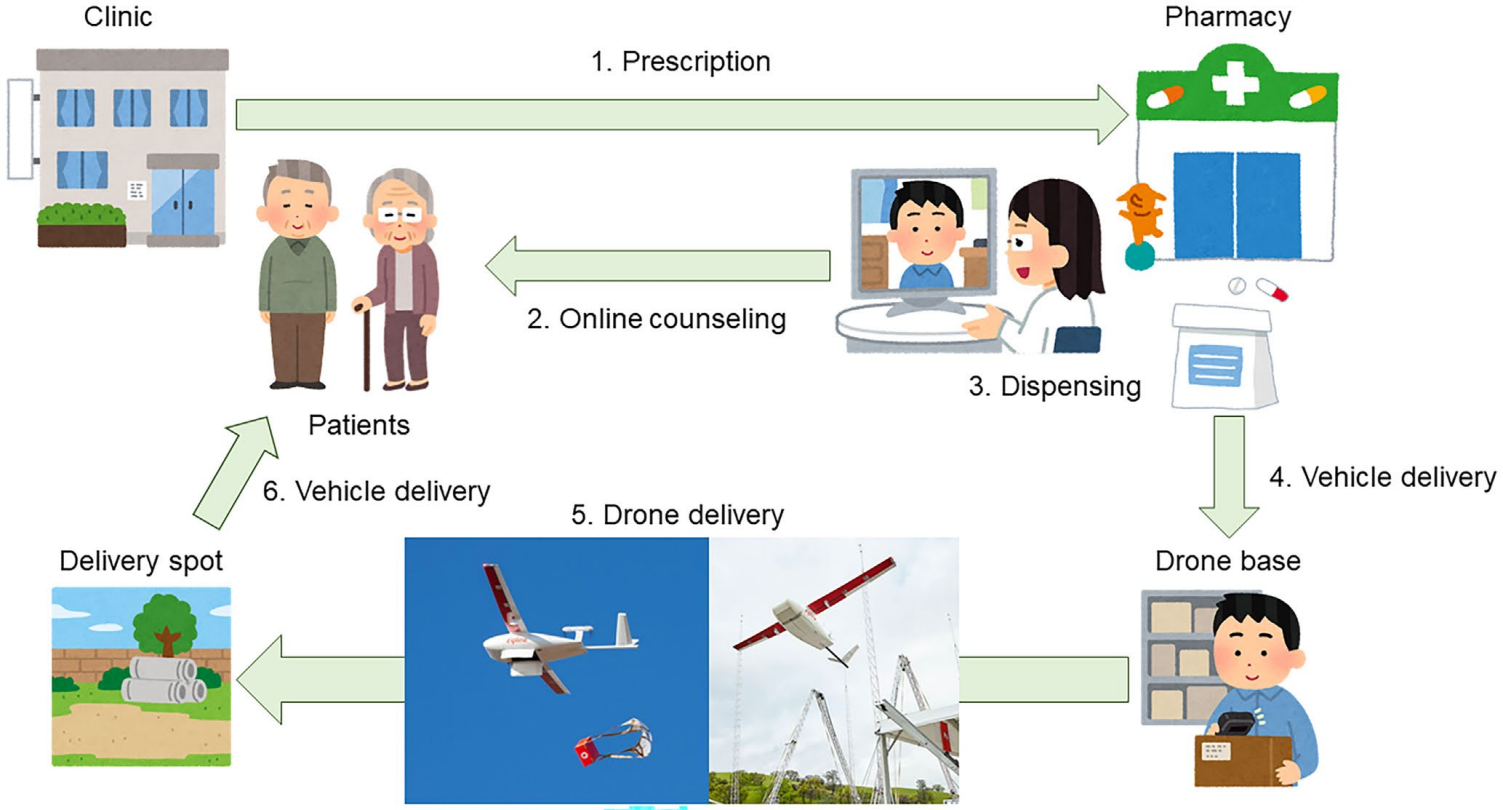
Flow of prescribing, online medication counseling, dispensing, and medication delivery.

The demonstration was performed according to the procedure illustrated in Figure 2. First, the prescription was sent to Fukue Pharmacy via a fax machine after a face‐to‐face examination by a physician in the clinic (Hisaka or Tamanoura Clinic). Second, pharmacists provided online medication counseling to patients in a private room in the clinic using the equipment we had prepared and connected to the Internet in advance. Patients returned home or went to work after counseling. Third, the pharmacists dispensed medications. The dispensed medications were stored in the pharmacy until their flight schedule. Fourth, the medications were delivered by a vehicle from Fukue Pharmacy to the drone base. The pharmacy and the base were approximately a 10‐minute drive away. Fifth, the dispensed medications were transported by autonomous fixed‐wing drones from Fukue District to Hisaka Island or Tamanoura District, dropped at a predetermined delivery spot on the shore, and picked up by the staff. Before dropping out, our staff confirmed the safety of the spot and signaled permission to drop the medications. The flight time from Fukue to Hisaka was ~10 min (13 km), and that from Fukue to Tamanoura was ~35 min (55 km) because of the detour along the northern coast of the island (Figure 1). In bad weather conditions that would interfere with flights (strong winds over 14 m/s, heavy rains over 50 mm/h, or cold or snowy weather), we intended to deliver medications by vehicle directly from the pharmacy instead of drones. Sixth, the medications were delivered by vehicle from the delivery site to the patients. The medications were visually checked for damage. The patient paid a fee to the pharmacy in exchange for the medication and received a cooperation fee. We then transmitted these fees to the pharmacy.

### 2.3 Drone delivery

An autonomous fixed‐wing drone delivery system established by Zipline, which was first developed for delivering life‐saving blood and medical supplies in Rwanda in 2016 and now operates drone logistics in seven countries as of October 2024, was utilized in this project.^18^, ^19^ Compared with other types, such as multi‐rotor, single‐rotor, and vertical takeoff and landing (VTOL) drones, fixed‐wing drones offer the advantages of flying faster and for longer periods, and they exhibit greater stability in windy conditions. However, they have the disadvantage of requiring a dedicated takeoff and landing base. After packing medications in a padded container with its own small parachute into a drone, the drone was fired into the air with a catapult, headed for the delivery spot using global positioning system (GPS) navigation, reached the spot, and dropped the padded container with its parachute. The drone then returned to the base, was recovered by an arresting‐hock‐assisted system, and was ready to fly again after a battery swap. In the unlikely event that the drone cannot continue flying, a parachute attached to the drone is deployed, allowing the drone to land gently.

At the destination, the receiver obtained a message that the drone reached and then came to the spot, confirmed the safety of the spot, and signaled permission to drop the medications. The drone computed the optimal drop timing based on the wind direction and speed around the delivery spot, allowing it to drop medications within a 10‐meter radius of the spot. It was programmed to circle the overhead and wait until the optimal timing was reached if the signal from the receiver at the delivery site was not obtained or if strong winds were blowing.

The drone used for this demonstration was called “Sparrow,” with an overall length of 1.9 m, a wing span of 3.3 m, and a maximum takeoff weight of 21 kg. When the battery was fully charged, the drone covered a 160 km roundtrip range (80 km each way) with a 1.75 kg payload at a cruising speed of 100 km/h. The operators could simultaneously control eight drones regardless of whether the destination was the same. It can fly in strong winds (up to 14 m/s) and heavy rains (up to 50 mm/h). However, it cannot fly in cold or snowy weather because its wings would freeze, interfering with its flight. Most drone delivery costs were fixed costs, including drone battery, maintenance, insurance, labor, and land costs at the drone base. The higher the number of drone deliveries, the lower the cost per flight.

To protect the medications from damage caused by falls or rainwater, the medications were packed in a padded container within a drone. Delivery of medications that require a cold chain is feasible using refrigerants.

### 2.4 Regulatory issues

We performed this demonstration under three Japanese legislations: the Act on Securing Quality, Efficacy and Safety of Products Including Pharmaceuticals and Medical Devices^9^ as of June 2023; the Civil Aeronautics Act^20^ as of June 2023; and the Guidelines for Drone Delivery of Medications^17^ as of March 2023.

According to the Act on Securing Quality, Efficacy and Safety of Products Including Pharmaceuticals and Medical Devices, pharmacists cannot deliver medications before providing medication counseling. Moreover, they had to verify that the patient had received the delivered medication.

Under the Civil Aeronautics Act, drones operated by the Sora‐iina Corporation could only fly over the sea and drop medications on the shore because flights beyond visual line‐of‐sight over inhabited areas (called “Level 4” drone flights in Japan) have not been certified. Additionally, drone delivery could not be performed immediately after online medication counseling because flight schedules were restricted from passing over vessels. The departure times for Hisaka‐bound drones were limited to four (10:10, 12:10, 15:10, and 16:50) as of October 3, 2023, and those for Tamanoura were limited to four (10:20, 11:20, 12:20, and 13:20) as of November 6, 2023. It is time‐consuming to obtain a certification for sightless flights over inhabited areas and vessels composed of a drone‐type certification and a specialized remote operator certification.

Under the Guidelines for Drone Delivery of Medications, we could not deliver narcotics, some psychotropic medications, or toxic medications such as some anticancer medications and cholinesterase inhibitors.

### 2.5 Questionnaire surveys

After medication delivery, we administered a questionnaire survey to the participants. The survey consisted of questions about online medication counseling, drone delivery of medications, and the current project. With a few exceptions, the questions offered five choices (1, negative; 2, rather negative; 3, neutral; 4, rather positive; and 5, positive). The questionnaire was self‐administered. However, if the participants had difficulty completing it, they were interviewed based on the questionnaire by our staff who delivered the medications from the delivery site to the patients. The proportion of respondents for each option was calculated for the multiple‐choice questions. For those who chose two options in one question, the proportion was calculated as if “0.5 persons selected each choice.”

Additionally, a self‐administered questionnaire survey was conducted with clinic and pharmacy staff. Questions regarding dispensing were added to the questionnaire administered to the clinic staff. The responses provided later were used for one of the clinic staff members who responded to the survey twice.

## 3 RESULTS

During the demonstration period, 62 patients (32 men) participated. We delivered medications directly from the pharmacy to two patients using a vehicle because strong winds interfered with drone delivery. It took more than 1 h for most patients to receive their medications after online medication counseling because of the limitations of flight schedules. In all drone deliveries, no in‐flight problems, medication dropping outside the delivery spot, or damage to dropped medications were observed. No participants complained about damage to their medication.

The medications delivered in this demonstration are listed in Table 1, according to the anatomical therapeutic chemical (ATC) classification. Most medications were used to treat chronic diseases, and few immediately desired medications (e.g., antibacterial) were delivered. No medications were difficult to deliver using drones because of their size or weight, including 420 tablets in total, 70 packets of powdered herbal medicine (2.5 g per packet), 150 g of ointment, 100 mL of topical analgesic, and 63 pieces of poultice for pain relief, and required cold chain delivery.

**TABLE 1:** The medications delivered in this demonstration according to anatomical therapeutic chemical (ATC) classification.

ATC classification	Routes	Number of types of medications	Number of patients prescribed
A: Alimentary tract and metabolism			
A01: Stomatological preparations	Cutaneous (for oral cavity)	1	1
A02: Drugs for acid‐related disorders	Oral	8	18
A05: Bile and liver therapy	Oral	1	2
A06: Drugs for constipation	Oral	3	6
A07: Antidiarrheals, intestinal anti‐inflammatory/anti‐infective agents	Oral	1	1
A10: Drugs used in diabetes	Oral	8	10
A11: Vitamins	Oral	2	5
B: Blood and blood‐forming organs			
B01: Antithrombotic agents	Oral	6	6
B02: Antihemorrhagics	Oral	1	1
B03: Antianemic preparations	Ocular	1	1
C: Cardiovascular system			
C01: Cardiac therapy	Oral	3	4
C03: Diuretics	Oral	2	3
C07: Beta‐blocking agents	Oral	3	6
C08: Calcium channel blockers	Oral	3	24
C09: Agents acting on the renin–angiotensin system	Oral	10	38
C10: Lipid modifying agents	Oral	3	22
D: Dermatologicals			
D04: Antipruritics, including antihistamines, anesthetics, etc.	Cutaneous	1	1
D07: Corticosteroids, dermatological preparations	Cutaneous	2	2
G: Genito urinary system and sex hormones			
G04: Urologicals	Oral	4	5
H: Systemic hormonal preparations, excluding sex hormones and insulins			
H03: Thyroid therapy	Oral	1	1
J: Anti‐infectives for systemic use			
J01: Antibacterials for systemic use	Oral	1	1
M: Musculo‐skeletal system			
M01: Anti‐inflammatory and antirheumatic products	Oral	1	1
M02: Topical products for joint and muscular pain	Oral	1	4
	Cutaneous	1	2
	Transdermal	2	29
M03: Muscle relaxants	Oral	1	1
M04: Antigout preparations	Oral	1	9
M05: Drugs for treatment of bone diseases	Oral	2	2
N: Nervous system			
N02: Analgesics	Oral	2	4
N03: Antiepileptics	Oral	1	2
N04: Anti‐Parkinson drugs	Oral	3	2
N05: Psycholeptics	Oral	1	2
N06: Psychoanaleptics	Oral	2	2
N07: Other nervous system drugs	Oral	1	1
R: Respiratory system			
R03: Drugs for obstructive airway diseases	Oral	1	1
	Inhaled	2	2
R06: Antihistamines for systemic use	Oral	1	1
S: Sensory organs			
S01: Ophthalmologicals	Ocular	4	8
Not classified by ATC classification^a^	Oral	6	8
	Ocular	1	1
	Cutaneous	1	2
	Transdermal	1	1

*Note*: Medications included in classifications L (antineoplastic and immunomodulating agents), P (antiparasitic products, insecticides and repellents), and V (various) were not prescribed.

^a^ Five of the oral medications were powdered herbal medicine packets.

**FIGURE 3:**
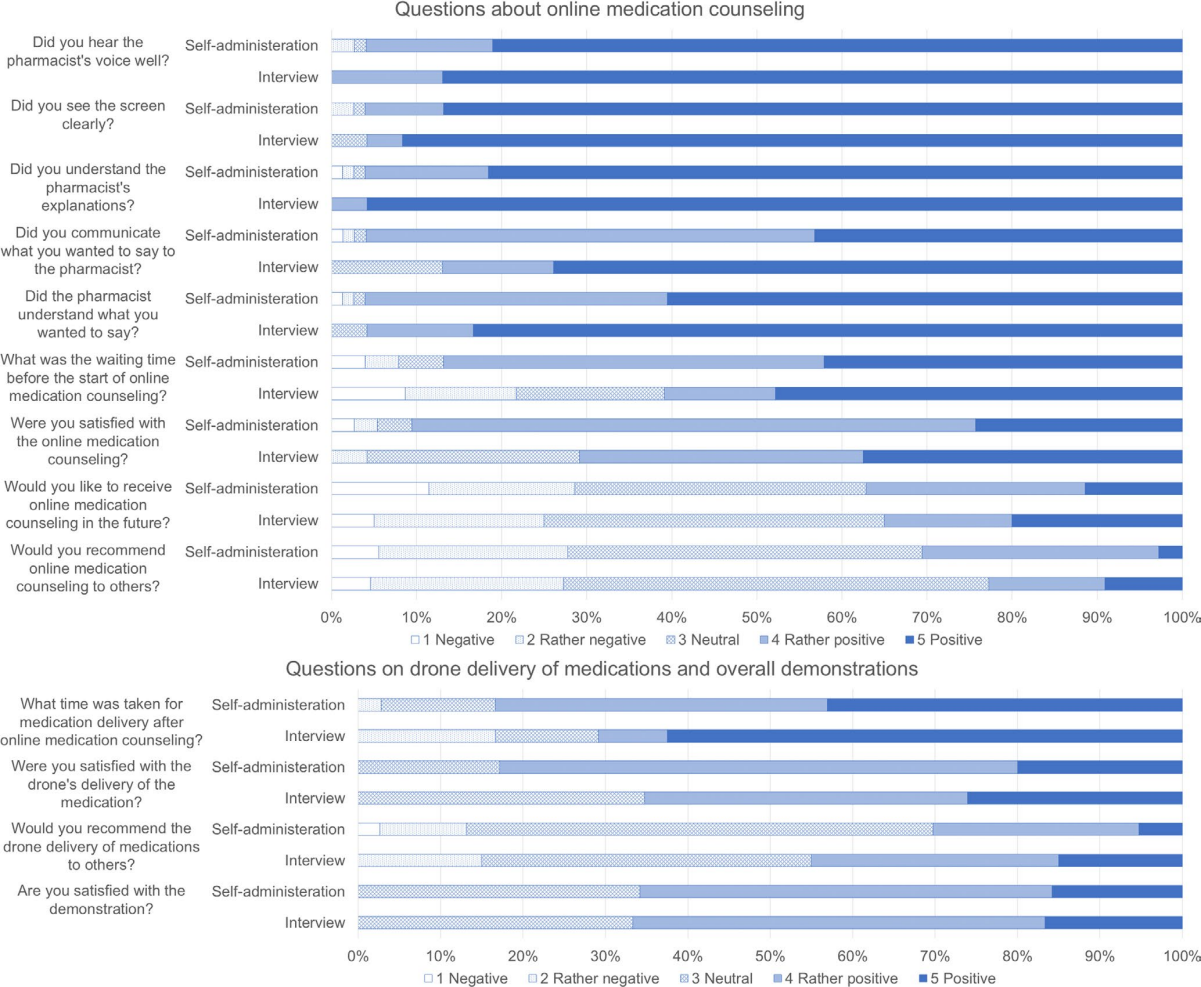
Results of the questionnaire survey for the patients for self‐administered and interview responses.

The results of the questionnaire to the patients are shown in Figure 3, which separates self‐administered and interview responses. All 62 patients responded (38 patients answered the questionnaire themselves and 24 patients were interviewed), ranging in age from 46 to 96 years (mean 77.3 years), and 21 lived alone. For all questions, the proportion of those who responded “positive” was higher for those who were interviewed (Figure 3). In the free‐response questions, four respondents reported that consulting a pharmacist was beneficial. In contrast, one respondent preferred face‐to‐face rather than online medication counseling. Regarding drone delivery and our project, eight respondents expressed that they would prefer to receive medications at the clinic, and three respondents expressed that they would accept if the public sector would promote a dispensing separation policy by using drone‐based medication delivery.

**FIGURE 4:**
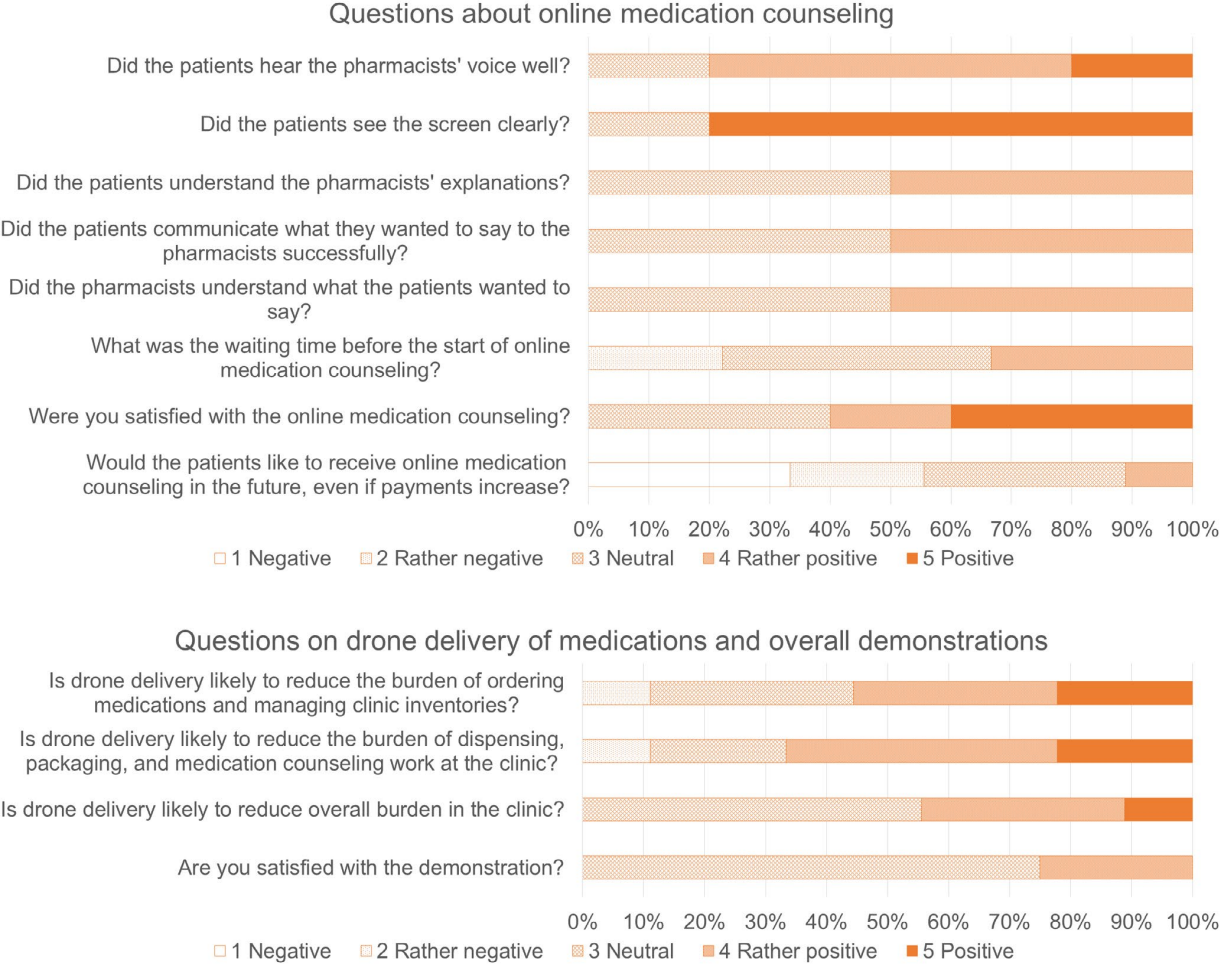
Results of the questionnaire survey for the clinic staff.

The results of the questionnaire administered to the clinic staff are shown in Figure 4. The respondents included one doctor, six nurses, and two clerical staff. Four clinic staff members responded that there were cases in which they had trouble with a few medications and six members responded that there were cases in which medications were in short supply. Three members responded that there were medication incidents. Regarding the future dispensing of medications, two respondents answered that it would be better to dispense them in the clinic every time, three respondents responded that they would occasionally dispense them outside with online medication counseling, and one respondent answered that they would dispense them outside the clinic every time. In the free‐response questions, two respondents expressed that pharmacists should provide information on medications, including how to take and store them. Regarding drone delivery and our project, three respondents expressed that our project would reduce the burden of ordering medications from wholesalers and managing inventory. Three respondents reported that it would assist in reducing dispensing and packaging work in clinics. However, two respondents expressed concerns about the patients' acceptance of this delivery system.

Two pharmacy staff members completed questionnaires. They reported not feeling burdened by this delivery system, including online medication counseling. However, they reported the need to assist patients in online counseling.

## 4 DISCUSSION

Prescribed medication delivery by autonomous fixed‐wing drones was technically feasible. Strong winds interfered with drone delivery in two cases but were limited. Most patients received their medications after more than an hour. Individual home delivery costs were involved because the delivery spot was restricted to the shore. We had to set up online medication counseling and handle patient payments to the pharmacy. Compared with medication delivery by drone elsewhere, our demonstration was novel in three aspects: (1) It was performed on multiple patients on the same day; (2) It spanned a three‐month period; and (3) It involved delivering the medication dispensed at the pharmacy on the same day. The medical service we demonstrated is applicable not only to remote islands but also to rural areas with low population density if our drones are allowed to fly toward these areas.

Although a few expressed a desire to receive their medications at the clinic, the patient survey results indicated that patients were delighted with online medication counseling. However, it should be noted that selection bias may have occurred because of the lack of specific criteria for selecting participants from the patients at Tamanoura Clinic, and positive skew bias, which indicates that respondents, particularly those who were interviewed, are likely to provide positive responses when answering questions about satisfaction. The clinic staff expressed that our project would reduce the workload regarding medications while worrying that patients would not be receptive to this delivery system. The pharmacy staff did not comment that they felt burdened by this delivery system, including the online medication counseling.

The project is expected to have the following effects on the healthcare system: (1) promotion of pharmacist participation in rural areas; (2) the expansion of patients' treatment options; (3) efficient allocation of pharmacists and pharmacies in areas where there is a shortage of pharmacists; (4) reduction in the work of ordering medications from wholesalers and managing inventory at clinics; (5) reduction in dispensing work in clinics; and (6) reduction in the cost of medications disposed of in clinics. First, separating prescribing and dispensing will reduce the misuse and overuse of medications, leading to patient safety and therapy effectiveness,^1^, ^2^, ^3^, ^4^, ^5^ as well as conflict of interest management.^1^, ^2^, ^3^ Second, patients in rural areas can choose from expanded treatment options because these medical facilities are limited in the number of medicines, especially in sparsely populated areas. Third, pharmacies can be consolidated while maintaining quality in areas where resources are limited. Fourth, the workload of the clinic staff related to ordering medications from wholesalers and managing inventory, which seemed significant, may have been reduced. Clinical staff will no longer have to devote their efforts to addressing medication shortages caused by supply disruptions. Fifth, pharmacies are more likely than clinics to automate dispensing, leading to safety and efficiency.

In addition to affecting the healthcare system, drone delivery has the potential to shift modes of transport for sustainable logistics and plays a key role in providing last‐mile parcel delivery with reduced carbon dioxide (CO_2_) emissions. In Japan, a projected shortage of truck drivers, estimated at 34% by 2030, may render the current logistics system unsustainable.^11^ Moreover, drones can contribute to a reduction in CO_2_ emissions compared with ground vehicles, particularly in areas with lower delivery density.^21^ Trucks account for most of the CO_2_ emissions in domestic logistics in Japan.^11^ Using drones for some last‐mile deliveries would help to address these problems.

It is important to choose the type of drone that best suits the characteristics and needs of the area in which it operates. Our fixed‐wing drones can fly faster and for longer periods, and we can operate multiple drones at the same time, even if they are headed to different destinations. In contrast, multi‐rotor and VTOL drones can take off and land in confined spaces but have a limited payload capacity and shorter flight durations. Single‐rotor drones can carry heavier payloads.

For the social implementation of drone delivery of medications in Japan, we need to address the following issues: (1) It is necessary to verify that the composition of medications does not change in various climates; (2) It is essential to perform sightless flights of autonomous fixed‐wing drones over inhabited areas and vessels; (3) It is necessary to consider who and how to confirm the safety of the delivery spot, signal the drones to permit the dropping of medications, and prevent medication theft and inappropriate use; and (4) It is necessary to consider who pays the delivery costs and how. First, some countries use cold chain delivery of medications, such as vaccines, by packing refrigerants in drones, and their effectiveness has been reported.^22^ However, further verification is required to apply this method in our setting, especially during the hot season. Second, if our drones can fly over inhabited areas and vessels, drone delivery can be performed away from the sea, such as near the clinic and the patient's home, and immediately after online medication counseling, without restrictions owing to the timetable of the vessels. It is essential to deliver emergently needed medications quickly. However, our drone flights have not received certification for operation beyond visual line‐of‐sight over inhabited areas under the Civil Aeronautics Act.^20^ Third, someone must stand by at the delivery spot to confirm safety and pick up the dropped medication. The clinic staff can check and monitor the spot and pick up the medication if it is near the clinic. Even if a patient's home is far from the clinic, healthcare providers can pick them up when combined with home‐visit care or mobile health clinic care. If healthcare providers deliver medications to the patient after dropping out, it will be possible to safely deliver medications that are currently restricted under the Guidelines for Drone Delivery of Medications (narcotics, some psychotropic medications, and toxic medications),^17^ which require the participation of pharmacists to ensure safety and may become dead stock in small clinics because of the limited number of patients who are candidates for them. When dropping medications into the yards of patients' homes without healthcare providers, it is necessary to consider how to check and monitor the delivery spot and receipt of medications. Fourth, it is essential to consider covering the costs of drone delivery. To implement the demonstrated service effectively, economic research is necessary to assess the cost‐effectiveness of the combined service of online medication counseling and drone delivery of medications, as opposed to the traditional method where medications are stockpiled and dispensed in clinics. Fixed costs constitute a significant portion of the expenses for drone delivery; therefore, an increase in the number of drone deliveries reduces the cost per flight. For service fees, delivery charges are not covered by social insurance, because additional billing for services not included in the fee schedule is prohibited under Japan's universal health coverage.^23^ If the fee is collected from the patients, co‐payment will increase, possibly leading to financial hardship and reluctance to receive healthcare. Residents, the government, and insurers must understand if public funds cover the cost.

In conclusion, delivering prescribed medications using autonomous fixed‐wing drones was technically feasible. This project will contribute to separating the prescribing and dispensing functions, promote pharmacist participation in rural areas, and expand patient treatment options. We found limitations in the time it took for patients to receive their medications and the cost of individual home delivery because the delivery spot was restricted to the shore. Methods for checking and monitoring the delivery spot, ensuring successful delivery, and covering the cost of drone delivery need further exploration.

## AUTHOR CONTRIBUTIONS

**Jun Miyata:** Conceptualization; Data Curation; Formal Analysis; Investigation; Methodology; Visualization; Writing – Original Draft Preparation. **Hironobu Tsuchiya:** Conceptualization; Funding Acquisition; Investigation; Methodology; Project Administration; Resources; Supervision; Visualization; Writing – Review & Editing. **Fumiaki Nonaka:** Conceptualization; Investigation; Methodology; Resources; Writing – Review & Editing. **Yuji Aso:** Conceptualization; Investigation; Methodology; Resources; Writing – Review & Editing. **Masanori Sugahara:** Conceptualization; Methodology; Writing – Review & Editing. **Takahiro Maeda:** Conceptualization; Funding Acquisition; Methodology; Project Administration; Supervision; Writing – Review & Editing.

## FUNDING INFORMATION

This research was supported by the Project on Promoting Healthcare Business Creation in Fiscal Year 2023 from the Ministry of Economy, Trade and Industry, Government of Japan. The funding source had no role in the design of this demonstration, conduct, data analysis, or decision to submit this manuscript for publication.

## CONFLICT OF INTEREST STATEMENT

Author Hironobu Tsuchiya is affiliated with Sora‐iina Corporation, a commercial drone company.

## ETHICS STATEMENT

The protocol for this demonstration was approved by the Ethics Committee of Nagasaki University Graduate School of Biomedical Sciences (project registration number: 23072804). This demonstration was conducted in accordance with the ethical standards of the 1964 Declaration of Helsinki and its subsequent amendments.

## CONSENT

All the participants provided written informed consent.

## CLINICAL TRIAL REGISTRATION

This demonstration was registered with the UMIN Clinical Trials Registry (UMIN000052074).

## Data Availability

An anonymous dataset supporting the findings of this demonstration is available from the corresponding author upon reasonable request.
